# Does owning improved latrine facilities enhance the safe disposal of child feces in Africa? a systematic review and meta-analysis

**DOI:** 10.1371/journal.pone.0303754

**Published:** 2024-05-16

**Authors:** Negasa Eshete Soboksa, Beekam Kebede Olkeba, Mekonnen Birhanie Aregu

**Affiliations:** 1 Department of Environmental Health, College of Health Sciences and Medicine, Dilla University, Dilla, Ethiopia; 2 Department of Environmental Health, College of Medicine and Health Sciences, Hawassa University, Hawassa, Ethiopia; Cranfield University, UNITED KINGDOM

## Abstract

**Introduction:**

Improved sanitation refers to those that effectively avoid human contact with excreta in a hygienic manner. Having improved latrines is a key factor in adopting safe ways of disposing of child feces. However, previous studies in Africa that examined how owning improved latrine facilities associated with household child feces disposal practices has shown inconsistent results, and no systematic review of these findings has been done. Therefore, this study aims to synthesize the evidence on the significance of households having improved latrine facilities for safe child feces disposal practices among households with under five-year-old children in Africa.

**Methods:**

The searched databases include: PubMed/Medline, Ovid/Embase, ScienceDirect, AJOL and the Cochrane Library. In the search process, Google Scholar and references of other studies were considered. This review included studies that were published in English without any time restrictions. The outcome of this study was an estimate of the association between the ownership of an improved latrine and the disposal practices of children’s feces. Two reviewers used the Excel data extraction tool to extract the relevant data from the studies that were included in the review. Using Stata version 16, a meta-analysis was performed with a random effects statistical model. The inverse index of variance (I^2^) was used to assess heterogeneity. Forest plots were used to show the pooled estimate with a 95% confidence interval. Publication bias was assessed using Egger’s test and a funnel plot.

**Results:**

Out of the 616 studies that were retrieved, 15 were included in the systematic review analysis and 10 were included in the meta-analysis. All studies that were included are cross-sectional studies done in Ethiopia, Nigeria, Gambia, Malawi, Eswatini, Ghana, Zambia, and a study used data from sub-Saharan Africa. Improved latrine facilities significantly enhanced the practice of safe child feces disposal, as shown by the overall effect size (OR = 2.74; 95% CI = 1.24–1.35, I^2^  =  99.95%). In the subgroup analysis by sample size, the presence of improved latrines significantly enhanced safe child feces disposal in studies with sample sizes less than 1000 (OR = 3.24; 95% CI = 2.86–3.62, I^2^  =  61.38%), while there was no significant difference in studies with sample sizes greater than 1000 (OR = 2.67; 95% CI = 0.69–4.64, I^2^  =  99.97%). However, studies that involved children under 5 years old indicated that improved latrine facilities significantly enhanced the practice of safe child feces disposal (OR = 4.02; 95% CI = 2.03–6.09; I^2^  =  99.96%).

**Conclusions:**

In this research study, we examined the ownership of improved latrine facilities among households with five-year-old children to enhance the disposal of child feces in a safer manner in Africa. The high heterogeneity among the studies and the cross-sectional design of the included studies limit the causal inference and generalizability of the findings. Therefore, meta-analyses of longitudinal and experimental studies are needed to confirm the causal relationship between improved latrine facilities and safe child feces disposal practices in Africa.

## Introduction

Sanitation services include the management of excreta from individual facilities, including the emptying and transport of excreta for treatment and eventual discharge or reuse. Improved sanitation facilities are defined as those that hygienically separate human waste from human contact. These include: flush or pour-flush to piped sewer systems; septic tank pit latrines; ventilated-improved pit latrines; pit latrines with slabs; and composting toilets [1]. A safe sanitation system is designed and used to isolate human excreta from human interaction at all stages of the sanitation service chain, from safe toilets and containment (in some in-situ treatment systems) through transportation (in sewers or by emptying and transportation), treatment, and final disposal or end-use [2]. Worldwide, more than 1.5 billion individuals lack access to fundamental sanitation facilities like personal toilets or latrines. Among them, 419 million continue to defecate in open spaces, such as street gutters, behind foliage, or directly into water bodies [3]. In Africa, Nigeria, Ethiopia, and Niger have the highest number of individuals engaging in open defecation, with 54 million, 43 million, and 15 million people, respectively. This indicates that these three nations have a significant prevalence of open defecation among their populations [4].

Many countries are challenged to provide sufficient sanitation to all their communities and put people at risk [5]. The lack of proper sanitation infrastructure can result in the contamination of the environment by fecal matter carrying infectious agents, increasing the risk of transmission to others. Each year, 827 000 people in low- and middle-income countries die as a result of inadequate water, sanitation, and hygiene [6]. Diseases like cholera, diarrhea, dysentery, hepatitis A, typhoid, and polio can spread because of poor sanitation [7]. It also has a significant impact on a number of neglected tropical diseases, including hunger, intestinal worms, trachoma, and schistosomiasis [6]. According to WHO reports on the burden of disease caused by unsafe drinking water, sanitation, and hygiene, globally, 69% of diarrhea cases, 14% of acute respiratory infections (ARIs), and 10% of under nutrition disease burden are attributed to unsafe WASH practices. Additionally, it is assumed that 100% of the disease burden from soil-transmitted helminthes (STHs) is attributable to unsafe WASH practices [8].

Waste from infected people can contaminate a community’s soil and water without sufficient sanitation facilities, raising the risk of infection for other people. The spread of many disease-causing pathogens can be slowed down by properly disposing of waste [9]. A cluster randomized trial study conducted in Odisha, India, found that while latrine coverage increased, rural sanitation programs did not change safe disposal habits [10]. However, the availability of improved latrines is a necessary condition for adopting safe child feces disposal methods [11,12]. To reduce open defecation, the majority of sanitation projects concentrate on providing latrine hardware and encouraging latrine use. Additionally, access to a latrine is frequently used to quantify open defecation, which may not accurately represent open defecation among small children. It has been demonstrated in the past that advances in sanitation have minimal effect on how children excrete and handle their excrement. [13,14]. Previous studies has revealed that having an improved latrine enhances the likelihood of safe child feces disposal practices in Ethiopia [15], South Africa [16] and Nigeria [17].

Previous research in African countries on the association between improved latrine facility ownership and household child feces disposal practices has yielded mixed results, with no attempt to conduct a systematic review of the findings. Therefore, the main goal of this study is to gather evidence on the significance of households having improved latrine facilities for the safe disposal of child feces and to produce findings that could support policy changes aimed at addressing public health concerns linked to the influence of owning improved latrine facilities on how households dispose of child feces in Africa. The review’s research question was, "Does owning improved latrine facilities enhance the safe disposal of child feces in Africa?"

## Methods and materials

### Study design and setting

A systematic review and meta-analysis were conducted to examine the association between ownership of improved latrine facilities among households with under five-year-old children and the disposal of child feces in Africa. It was conducted following the preferred reporting items for systematic review and meta-analysis [18] (S1 Checklist).

### Eligibility criteria

Studies reporting the association between owning an improved latrines facility and child feces disposal practices among households with under five-year-old children in African countries were included in this systematic review and meta-analysis. Published research publications and unpublished studies, including preprints and gray literature written in English, were all eligible regardless of publication date or study duration. The analysis also included all studies that used any type of study design and reported the association between improved latrine ownership and child feces disposal practices.

### Search databases and strategy

The searched databases include: PubMed/Medline, Ovid/Embase, ScienceDirect, AJOL and the Cochrane Library were searched. In the search process, Google Scholar and references of other studies were considered. The initial step involved conducting a preliminary search using medical subject headings (MESH terms). Next, keywords were created based on the key terms found in the articles from the initial search. Subsequently, both MESH terms and keywords were utilized to search for articles in databases as well as other search engines (S1 Table). Additionally, input from librarians was sought to locate unpublished research related to the topic of interest for the systematic review and meta-analysis. Search terms were developed following the PEO guidelines [19]. Articles were sought by utilizing MeSH terms and keywords in online databases. Boolean operators such as "AND" and "OR" were employed to connect MeSH terms and keywords during the search process. Search terms developed and used in this analysis were "sanitation," "toilet facilities, "improved latrine facilities," ""ownership," "child," "feces," "safe disposal," "waste disposal," "fluid," and "Africa."

### Quality of included study assessment

Two reviewers (NES and BKO) assessed the methodological quality of the articles chosen for retrieval using the standardized critical appraisal instruments from the Joanna Briggs Institute Meta-Analysis of Statistics Assessment and Review Instrument before including them in the review (JBI) [20] independently. Any discrepancies were resolved through conversation or with the help of a third reviewer (MBA).

### Outcome of measurement

The outcome of this study was an estimate of the association between the ownership of an improved latrine and the disposal practices of children’s feces. Improved latrines and children’s feces disposal practices were defined based on the WHO/UNICEF-JMP on water supply and sanitation guidelines [21]. Improved latrines are those that separate human waste from human contact, such as flush or pour-flush systems, pit latrines with ventilation, or composting toilets. If the respondents have this type of latrine, we consider them to have improved latrine, unless they have unimproved. Children’s feces disposal practices were classified as safe disposal being when feces were collected and disposed of in a latrine or buried, and unsafe disposal being when feces were put down a drain or ditch, thrown away, or left in the open.

### Data extraction and synthesis

Before beginning data extraction, identified articles were imported into Mendeley Desktop to identify and remove duplicates. Using the modified data extraction tool from JBI, the necessary information was gathered from the records included in the review independently by two authors (NES and BKO). The information that was retrieved comprises specific information about child feces disposal practice, population included in the study, study methods, type of latrine they owned and results that were pertinent to the review topic and intended objectives.

The review was pooled in a statistical meta-analysis utilizing Stata 16. Double data entry was applied to all outcomes. The study examined the link between owning improved latrine facilities and how households dispose of child feces. The findings were presented as odds ratios with 95% confidence intervals. To assess the variation among studies, the Cochrane Q test and I^2^ statistics were used. The I^2^ statistics measure the degree of variation within the studies included, with values ranging from 0 to 100%. Values 0–25% indicate minimal heterogeneity, 25%–50% indicate low heterogeneity, 50–75% indicate moderate heterogeneity, and values 75%–100% indicate significant heterogeneity among the studies [22]. The restricted maximum likelihood random effect model was used to estimate the pooled association between improved latrine facility ownership and household child feces disposal practices. A random effect model was employed due to high heterogeneity (I^2^ = 99.95%). A forest plot was used to illustrate the pooled association between improved latrine facility ownership and household child feces disposal practices with a 95% CI. A forest plot was used to display the combined association, and publication bias was assessed visually and statistically using Egger’s regression test. Sensitivity analysis was conducted to evaluate individual study impacts, and sub-group analysis based on study sample size and included age group was performed to compare them.

## Results

### Study selection procedure

**Fig 1:**
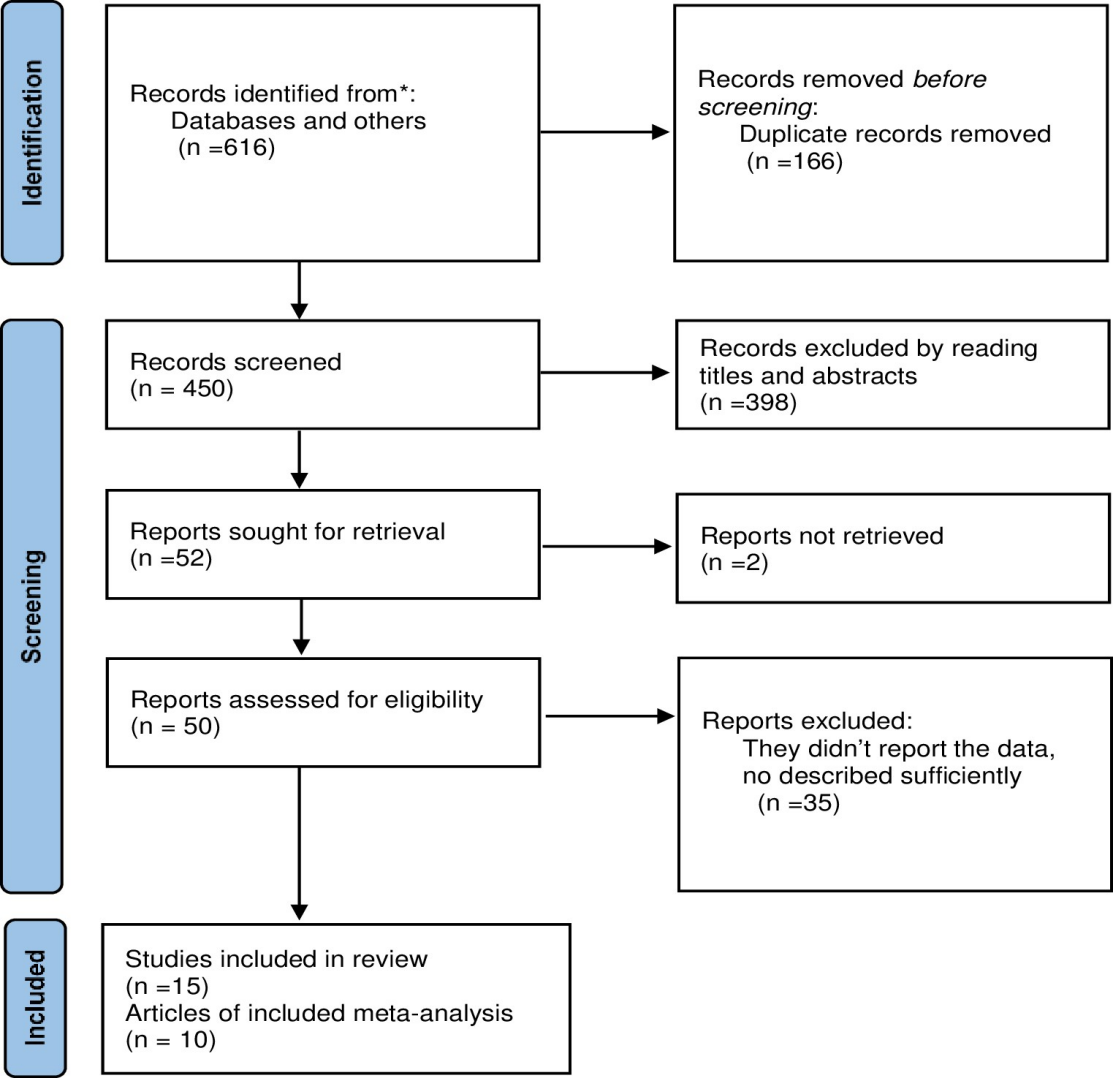
Flowchart of study selection for systematic review and meta-analysis of the influence of improved latrine facility ownership on household child excreta disposal behaviors.

In a total of 616 published studies were retrieved and from these 15 cross-sectional studies that met inclusion criteria and reported about the influence of improved latrine facility ownership on household child excreta disposal behaviors were included in this study [15,23–36]. In process due to overlap about 166 studies were removed from the records. After removing duplication, 450 studies were searched and then 398 additional articles were removed by reading the titles and abstracts. The remaining 52 articles were suitable for full-length article assessment, and we retrieved 50 of them; the remaining two articles were removed since we could not access the full-length article. Following the complete article read, 35 articles were excluded for the reasons stated. Finally, in this analysis 15 studies were included in the systematic review and 10 studies were included in the meta-analysis. Five studies were excluded from the meta-analysis due to inadequate reporting of essential data, despite their relevance to our research question (Fig 1).

### Included study characteristics

The characteristics of the included studies in this analysis are shown in Table 1. All included studies are cross-sectional studies conducted in Ethiopia, Nigeria, Gambia, Malawi, Eswatini, Ghana, Zambia, and a study used data from sub-Saharan Africa. Regarding the sample size, 301782 study participants were included in this analysis, which is the 128096 maximum sample sizes, whereas 300 is the smallest sample size of the included studies. From the total of 15 studies, 10 were considered under five children, four were done on under two-year-old children, and the remaining was a study done on under three-year-old children. Reports on the prevalence of safe child feces disposal (CFD) in studies were also reported in each study and the maximum and minimum prevalence reported were 85.6% and 19.7%, which practiced in day time, respectively (Table 1).

**Table 1:** Characteristics of included studies in this systematic review and meta-analysis.

S.NO	Author, publication year	Country	Study design	Sample size	Age includes	Prevalence of Safe CFD practices (%)	Study finding about safe CFD practices and latrine ownership
1.	Ayele *et al*. 2018 [35]	Ethiopia	Cross-sectional	445	<5	65.20	The presence of a functional latrine increases safe child feces disposal practices before adjustment for confounders.
2.	Addis *et al*. 2022 [33]	Ethiopia	Cross-sectional	888	<5	37.85	Households with basic sanitary facilities were more likely to conduct safe child feces disposal.
3.	Soboksa *et al*. 2021 [36]	Ethiopia	Cross-sectional	756	<5	67.78	Households with unimproved latrines were less likely to practice safe child feces disposal.
4.	Aluko *et al*. 2017 [25]	Nigeria	Cross-sectional	300	<5	19.7 (day) 69.0 (night)	Caregivers of children under the age of five who practiced safe sanitation were wealthy, and knowledge was substantially associated with ownership of household toilets.
5.	Beardsley *et al*. 2021 [23]	Ethiopia, India, and Zambia*	Cross-sectional	3737	<5	40.0 (Ethiopia) 54.0 (Zambia)	The odds of safe child feces disposal were higher in households with upgraded toilet facilities.
6.	Sahiledengle 2019 [24]	Ethiopia	Cross-sectional	4145	<5	36.9	Households having improved latrine facilities do not use them to dispose of child feces.
7.	Azage and Haile 2015	Ethiopia	Cross-sectional	11126	<5	33.68	Having access to an improved latrine increases safe child feces disposal practices
8.	Aliyu and Dahiru 2019	Nigeria	Cross-sectional	19288	<5	59.4	Unimproved toilet types risk safe child feces disposal methods.
9.	Sahiledengle 2020 [27]	Ethiopia	Cross-sectional	40520	<5	22.3	The likelihood of disposing of child feces in an unsafe manner were lower in households with improved toilet facilities than in households without such facilities.
10.	Nkoka 2020 [28]	Malawi	Cross-sectional	6326	<2	85.6	Women from households that had improved latrine facilities were more likely to dispose of their children’s feces safely.
11.	Simelane *et al*. 2020 [29]	Eswatini	Cross-sectional	2765	<3	58.2	Households with no toilet facility were more likely to dispose of child feces in an unsafe manner compared to households with a flush toilet.
12.	Seidu *et al*. 2021 [30]	sub-Saharan Africa	Cross-sectional	128096	<5	58.73	Respondents from families with better latrine facilities were more likely to dispose of their children’s feces safely.
13.	Tsegaw *et al*. 2023 [31]	Gambia	Cross-sectional	3011	<2	56.3	Households with an improved latrine were more likely to dispose of children’s waste safely, although not significantly.
14.	Demissie *et al*. 2023 [32]	sub-Saharan Africa	Cross-sectional	78151	<2	51.2	In comparison to their counterparts, respondents who reported not having access to latrines were more likely to practice safe child feces disposal.
15.	Seidu 2021 [34]	Ghana	Cross-sectional	2228	<2	24.5	Children’s feces were disposed of more safely by respondents from homes with improved latrines than by those without.

*Only data from Ethiopia and Zambia was used in this review.

### Included study quality

Before being included in the review, each original study’s quality was assessed using the Joanna Briggs Institute Meta-Analysis of Statistics Assessment and Review Instrument (JBI). Our analysis revealed that, out of the 15 included studies, 86.7% had a low risk of bias [15,23,24,26–36] and the remaining 13.3% had a moderate risk of bias [25,37] (S2 Table).

### Improved latrine influence on child feces disposal meta-analysis

**Fig 2:**
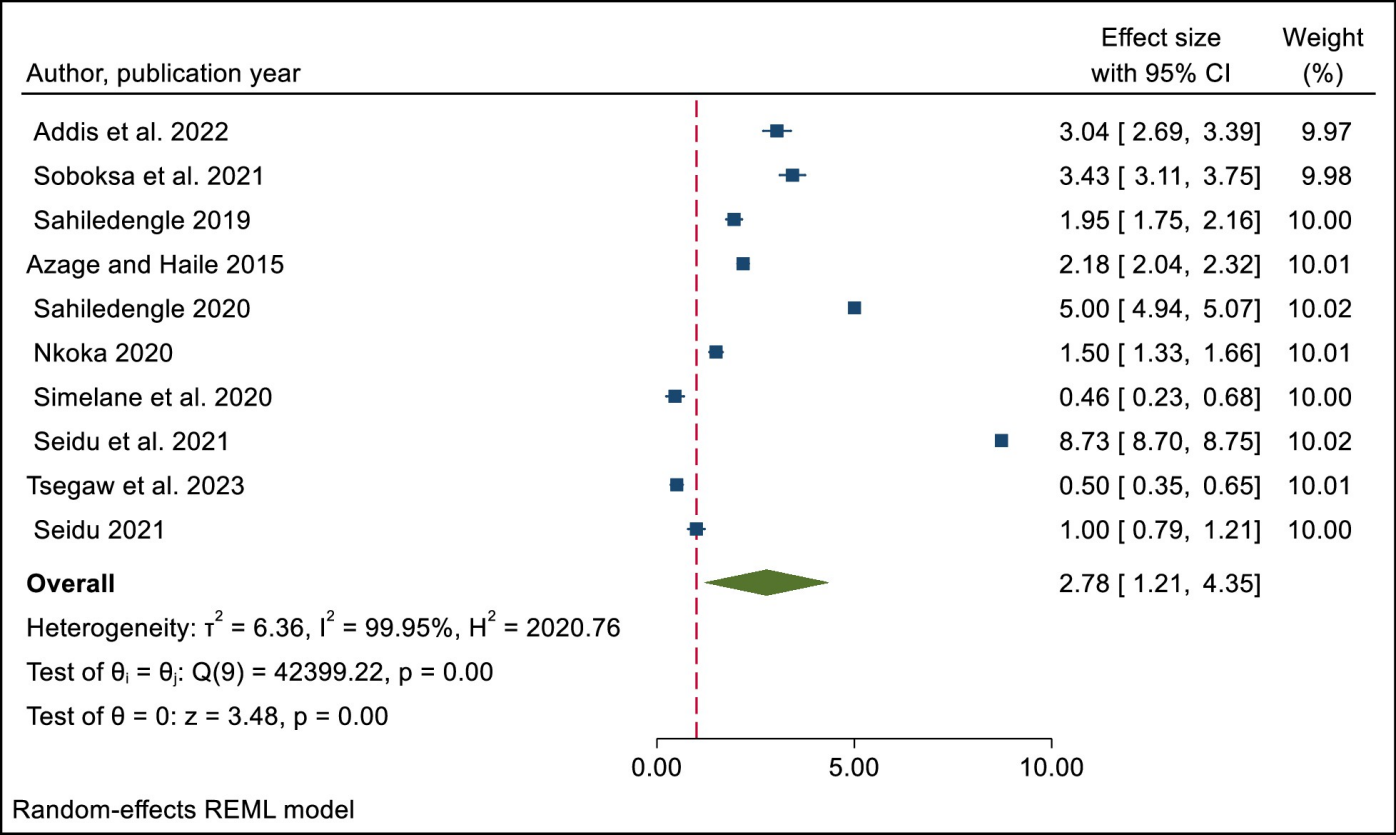
The overall pooled effect, size of study selection for systematic review, and meta-analysis of the influence of improved latrine facility ownership on household child excreta disposal behaviors.

Fig 2 shows the results of a meta-analysis of 10 studies on the effect of improved latrine facilities on safe child feces disposal practices. The figure indicates that respondents with improved latrine facilities were 2.78 times more likely to dispose of child feces safely than those without, as shown by the overall effect size (OR = 2.78; 95%CI = 1.21–4.35). The meta-analysis also showed high heterogeneity among the studies that were included in the synthesis (Fig 2).

### Subgroup analysis

**Fig 3:**
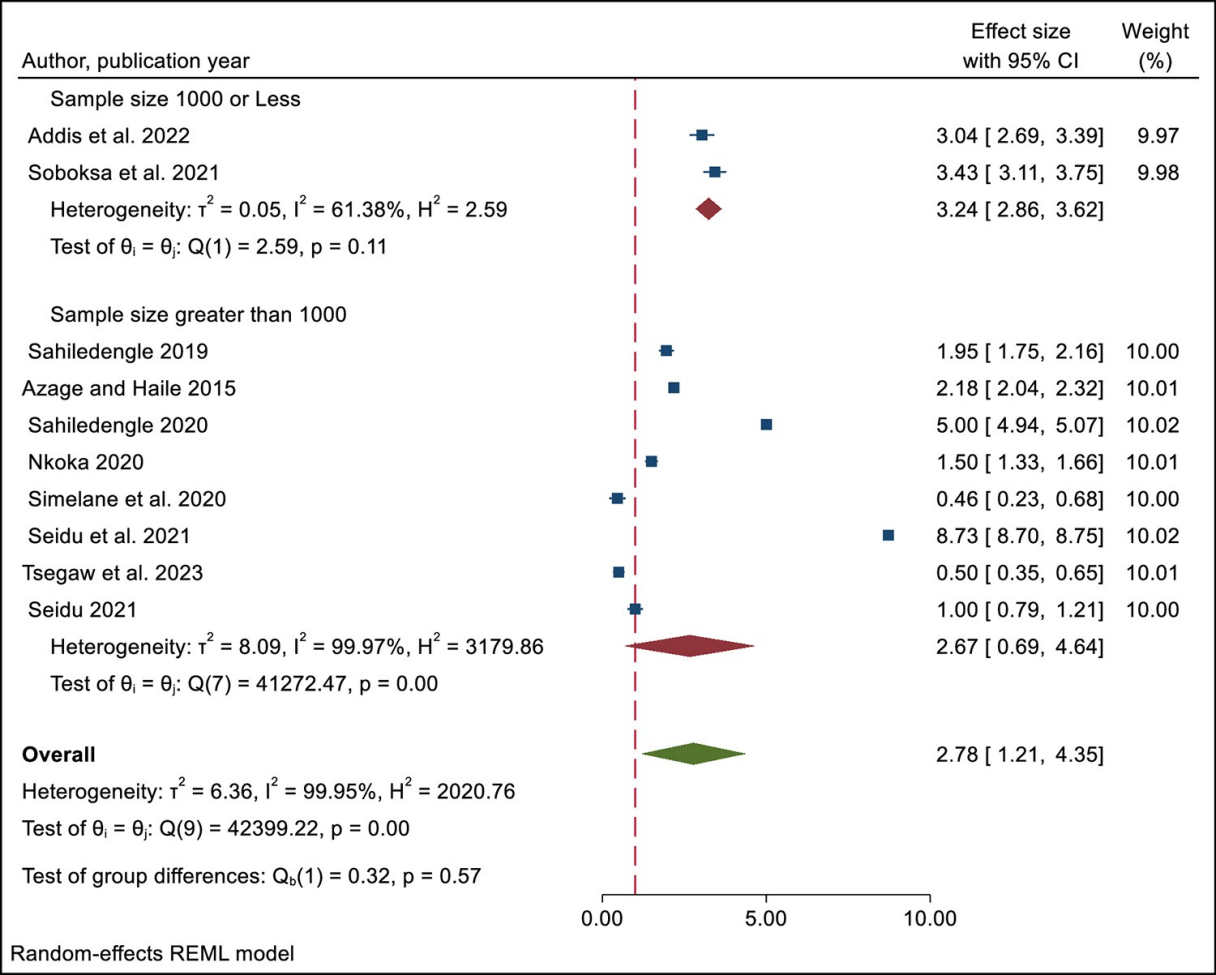
Subgroup analysis by sample size of meta-analysis of the influence of improved latrine facility ownership on household child excreta disposal behaviors.

Subgroup analysis was done by considering the sample size and age of the study subjects. We found that in studies with less than 1000 sample sizes, people who had improved latrines were more likely to dispose of their children’s feces safely (OR = 3.24; 95% CI = 2.86–3.62), compared to people who did not have improved latrine. However, in studies with more than 1000 sample sizes, there was no significant difference between the two groups (OR = 2.67; 95% CI = 0.69–4.64). This means that the sample size might have influenced the results of the meta-analysis. The analysis showed that there was heterogeneity among the studies that were included in the synthesis in both studies, with sample sizes less than 1000 (I^2^  =  61.38%) and greater than 1000 (I^2^  =  99.97%) (Fig 3).

**Fig 4:**
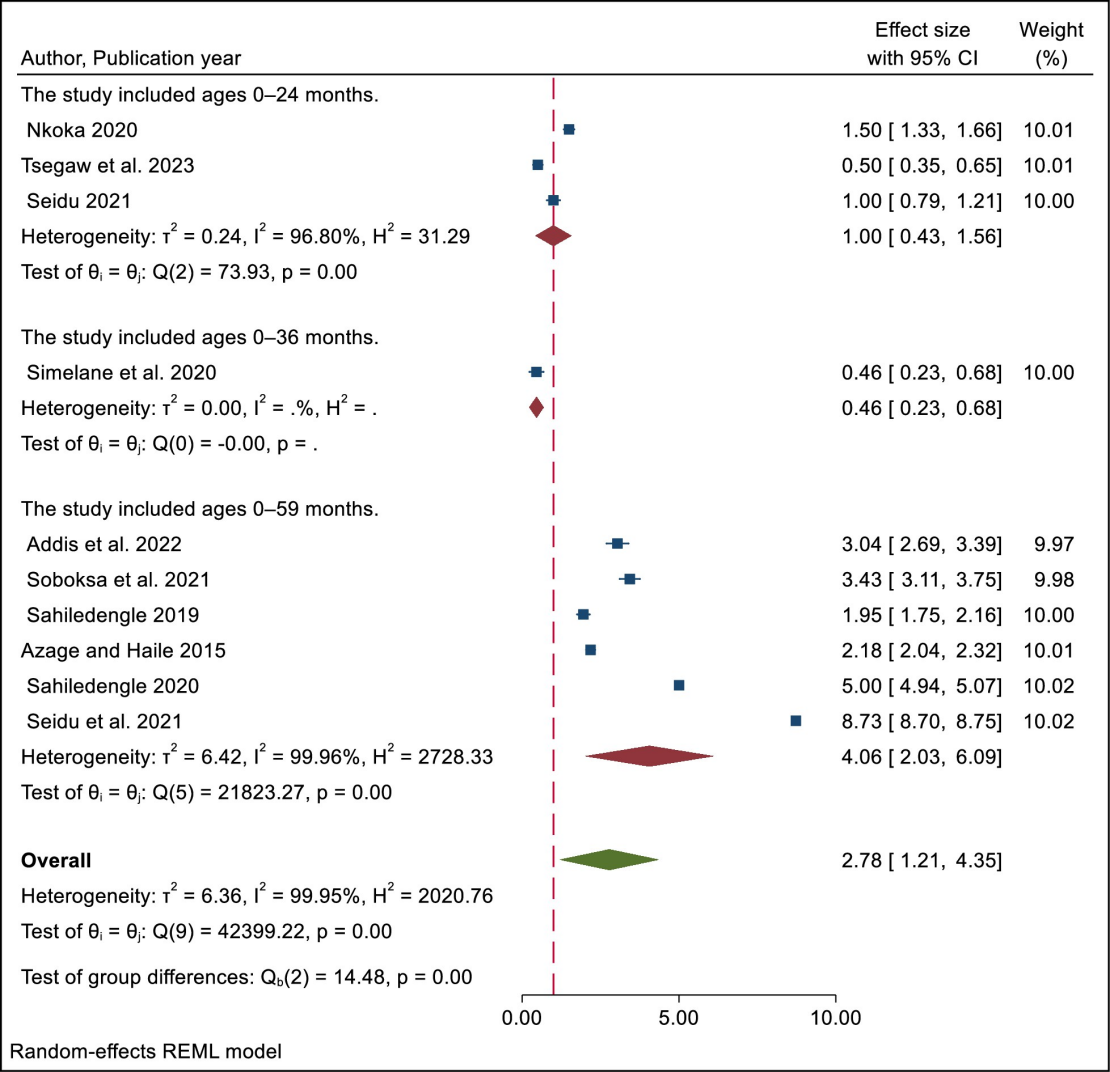
Subgroup analysis by study participant age of meta-analysis of the influence of improved latrine facility ownership on household child excreta disposal behaviors.

We also looked at how improved latrine facilities affected the way people disposed of their children’s feces based on the age of the children. We found that in studies that involved children under 5 years old, people who had improved latrines were more likely to dispose of their child’s feces safely (OR = 4.06; 95% CI = 2.03–6.09), compared to people who did not have improved latrine. However, there was also high heterogeneity among the studies in this group (I^2^  =  99.96%), which means that the studies were not very consistent or similar to each other. This might have affected the reliability of the meta-analysis (Fig 4).

### Publication bias

**Fig 5:**
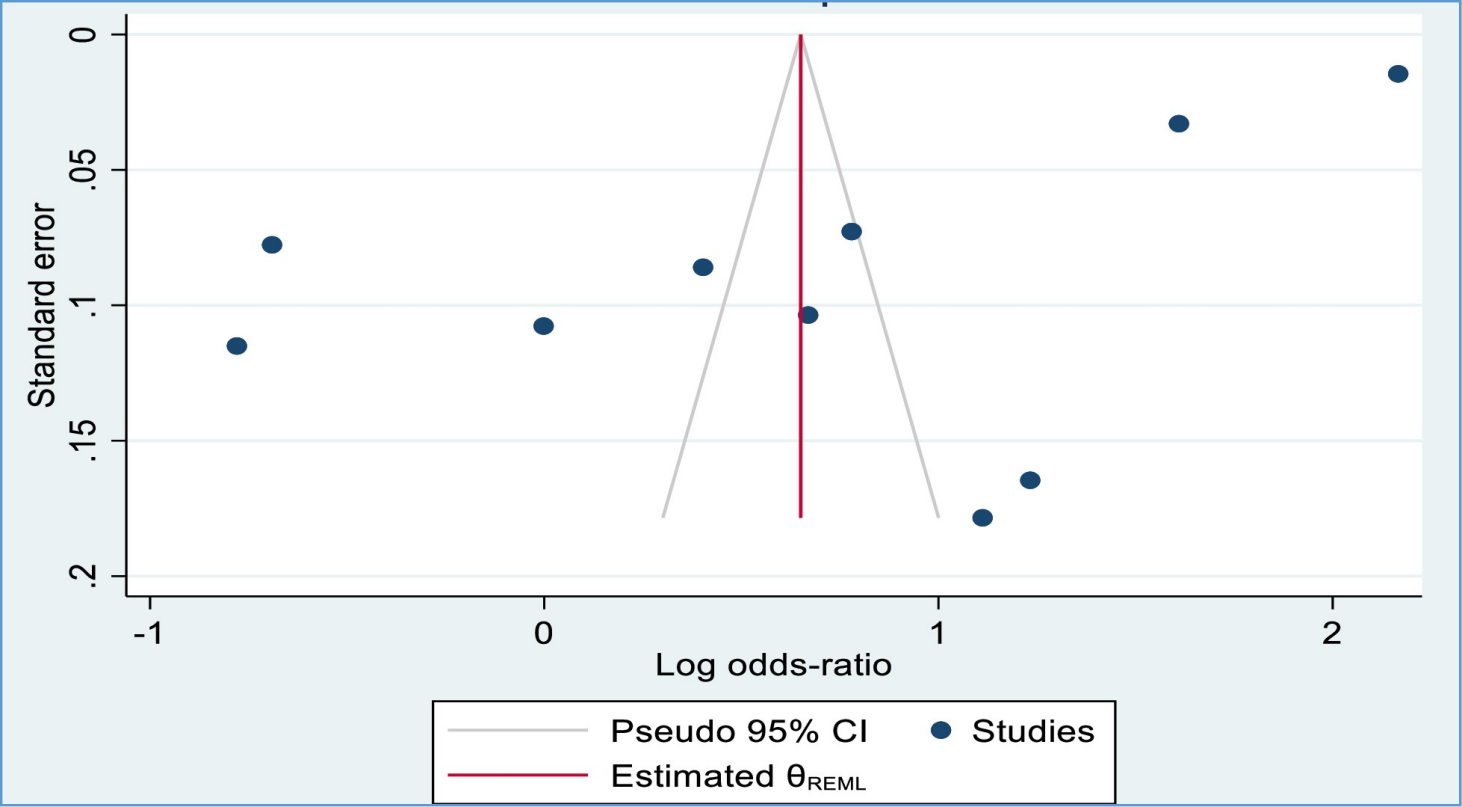
A funnel plot illustrating the effect of latrine facility ownership on household child excreta disposal behavior.

Fig 5 shows the funnel plot of meta-analysis of 10 studies that measured the association between improved latrine facilities and safe child feces disposal practices in Africa. The funnel plot appears to be asymmetrical, as there are more studies on the right side than on the left side, suggesting that there might be publication bias favoring studies with positive results. On the other hand, the results of the Egger’s test analysis show that the p-value is 0.012 and, thus, publication bias in the data that were included in this meta-analysis. This means that studies with positive or significant results were more likely to be published than studies with negative or non-significant results, which can affect the overall effect size estimate of the meta-analysis (Fig 5).

### Sensitivity analysis

**Fig 6:**
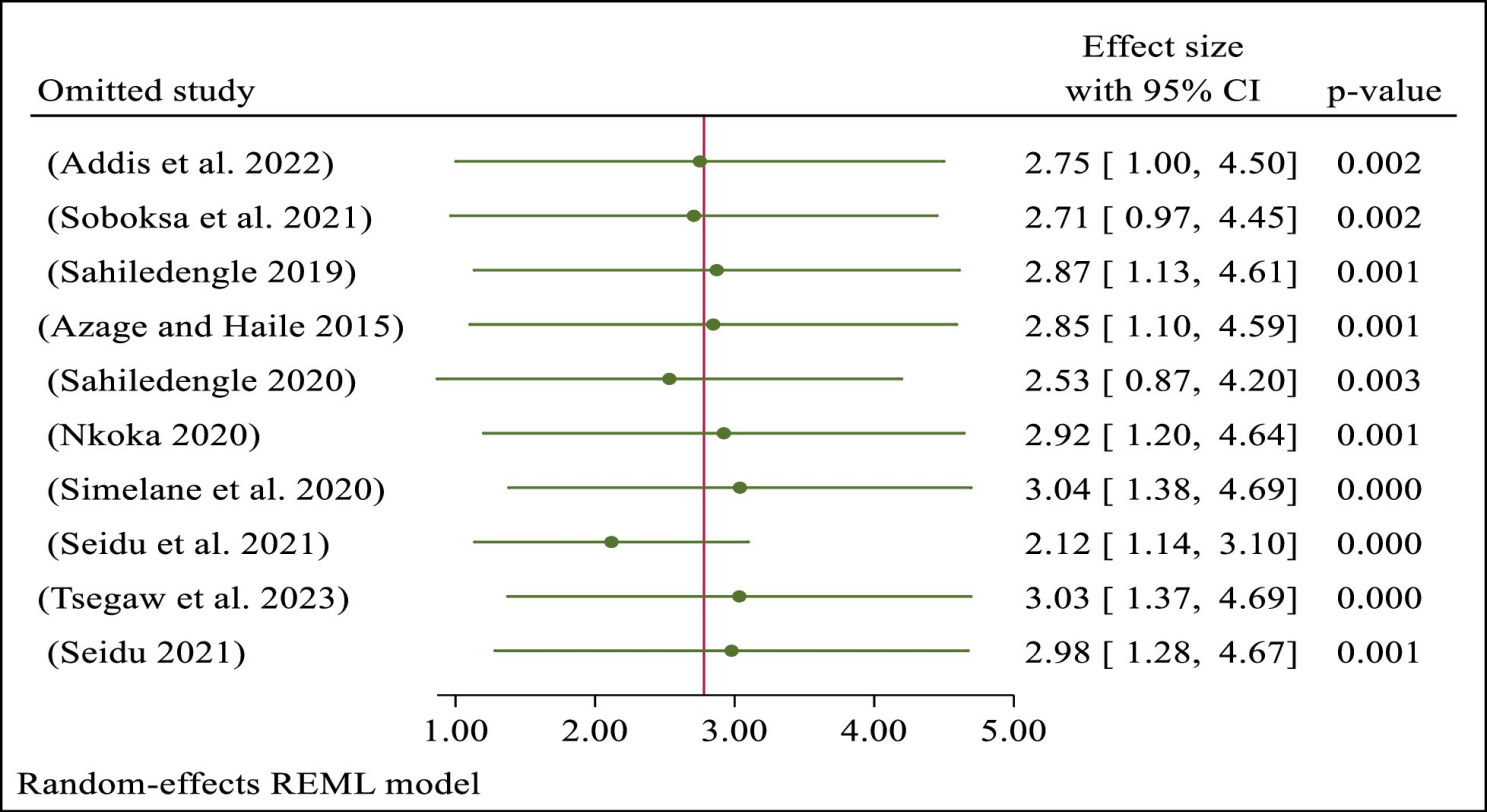
A sensitivity analysis of the included studies of the effect of latrine facility ownership on household child excreta disposal behavior.

As shown in Fig 6, the meta-analysis was performed as a sensitivity analysis of the studies. The sensitivity analysis revealed that the overall effect size, which reflects the impact of owning a latrine facility on how household children dispose of their excreta, was not significantly altered by any single study (Fig 6).

## Discussion

The current study was set out with the aim of combining the evidence on the importance of households having better latrine facilities for safe child excrement disposal behavior in Africa. It is commonly acknowledged that one of the most important public health interventions to stop the spread of infectious diseases is the use of improved sanitation for disposing of waste [38]. The review findings indicate that in Africa, there were significant relationships between households possessing improved latrine facilities and the implementation of safe child excreta disposal practices. This underscores the importance of improved latrine facilities in influencing positive behavior towards the safe disposal of child excreta.

The effective use of latrines for excrement disposal has the potential to significantly reduce the incidence of tropical diseases that are often overlooked, especially those that spread through the soil and are waterborne [39]. Diseases like trachoma, schistosomiasis, and soil-borne helminths are of particular importance. These can all be considerably decreased by using latrines appropriately and disposing of waste properly. This emphasizes the crucial role that proper sanitation facilities and behaviors play in safeguarding public health in Africa [40,41]. It also increases maternal and childhood morbidity and mortality, which in turn impacts the economic development of the country [38].

The majority of the studies included in our analysis reported prevalence rates, indicating that more than half of the participants practiced safe child feces disposal. This finding is encouraging, as it suggests that a significant proportion of caregivers are adopting appropriate hygiene practices when it comes to managing child feces. When comparing our findings to data from the Asia-Pacific region, where rates of safe feces disposal are reported to be less than 50% [42], it becomes evident that our study’s results are relatively better. This difference could be attributed to various factors, including differences in cultural norms, access to sanitation facilities, and awareness campaigns promoting safe hygiene practices [28].

The analysis of the included studies revealed a wide range of prevalence rates for safe child feces disposal (CFD), with the maximum and minimum reported rates being 85.6% and 19.7%, respectively. This variability in prevalence rates underscores the importance of understanding the factors influencing safe CFD practices in different populations and settings. Specifically, from the current included study, only 19.5% of safe child feces disposal practices are commonly practiced in Nigeria during the day. In contrast, Malawi has the highest prevalence of safe child feces disposal practices, with 85.6% of households following proper disposal methods. The study found that women in Malawian households with improved latrine facilities were more likely to dispose of their children’s waste properly. Even so, open disposal was still common in homes with toilets. According to a systematic review conducted in the Asia-Pacific area, these results point to the importance of putting large-scale programs and hardware interventions into place in order to give communities the tools they need to modify their habits and behaviors in the presence of improved latrine [43].

This study found that households with improved latrine facilities were more likely to practice safe child feces disposal as the children’s age increased. The results emphasize the importance of enhancing latrine facilities and promoting safe feces disposal practices, especially in households with older children, to reduce the risk of fecal-oral diseases and improve community health [38,44]. In our study, the subgroup analysis within studies that included children under the age of 5 found that there was a greater likelihood of feces being disposed of safely in households with improved latrines. Even though evidence suggests that children’s feces may pose a greater risk than adult feces because they are more likely to contain diarrhea-causing pathogens such as hepatitis A, rotavirus, and E. coli [45], this finding may be attributed to the belief that older children’s feces are more harmful than those of younger children, as well as containing more visible food residuals. As a result, there is a greater emphasis on the proper disposal of feces from older children, leading to higher odds of safe disposal in households with improved latrines [46].

A number of limitations should be considered when interpreting the results of this systematic review and meta-analysis. Firstly, the review was limited to publications written in English, which may have excluded pertinent studies published in other languages. Secondly, all of the studies included in the review had a cross-sectional design, which means that other confounding variables may have affected the outcome variable. Lastly, the review contained a small number of articles from a small number of African countries, which may limit the findings’ generalizability.

The study’s findings have significant policy implications for enhancing safe children’s feces disposal practices throughout African countries. The results emphasize the necessity of focused efforts, especially for households with young children, to encourage the use of improved latrine facilities. Behavior change communication, caregiver training programs, and the supply of reasonably priced and easily obtainable technology for secure disposal of child excrement should be the main focuses of initiatives. Additionally, in order to enhance general health outcomes and lower the risk of fecal oral illnesses, efforts should be made to increase the accessibility and availability of improved latrines in communities. It is recommended that policymakers give priority to funding, sanitary infrastructure, and hygiene education in order to enhance the safe disposal of child feces and promote healthy communities.

The study’s findings have also important implications for future research and practice aimed at improving safe children’s feces disposal practices in African countries. The results of the study indicate a number of avenues for additional investigation and real-world implementation. First of all, more investigation is necessary to gain a deeper comprehension of the particular elements that mediate the relationship between enhanced latrine facilities and responsible fecal disposal practices. This may involve investigating socio-cultural, economic, and behavioral determinants that influence the utilization of improved latrine facilities for child feces disposal. Studies should also be conducted to determine how better sanitation infrastructure will affect public health outcomes over the long run, such as a decline in the prevalence of soil- and water-transmitted illnesses. Regarding practical applications, the results highlight the necessity of focused interventions and educational initiatives meant to encourage appropriate use of improved latrine facilities for the secure disposal of child feces, particularly in homes with young children. Tailored educational initiatives, community engagement, and the development of age-specific educational materials may be effective strategies to encourage the adoption of safe child feces disposal practices. Additionally, it would be beneficial to explore the differential impacts of improved toilet facilities in diverse demographic and geographic contexts to inform the design of context-specific interventions and policies.

## Conclusions

In this research study, we examined the ownership of improved latrine facilities among households with five-year-old children to enhance the disposal of child feces in a safer manner in Africa. According to the subgroup analysis, improved toilet facilities increased the safe disposal of child feces in studies including children under five years old and small sample sizes. The cross-sectional design of the included studies and the high heterogeneity among them restrict the capacity to draw conclusions about causality and generalize the results. Therefore, in order to verify the causal association between better latrine facilities and safe child feces disposal practices in Africa, more longitudinal and interventional research meta-analyses are required.

## Supporting information

S1 Checklist PRISMA 2020 checklist. (DOCX)

S1 Table Search strategy for the PubMed database. (DOCX)

S2 Table Individual study quality was included in the analysis of this systematic review and meta-analysis. (DOCX)

## References

[1] WHO/UNICEF. Sanitation | JMP. 2021 [cited 24 Mar 2022]. Available: https://washdata.org/monitoring/sanitation.

[2] World Health Organization. Water, sanitation, hygiene and health: a primer for health professionals. Geneva; 2019. WHO/CED/PHE/WSH/19.149.

[3] WHO. Sanitation. In: WHO Key facts [Internet]. 2023 [cited 3 Mar 2024]. Available: https://www.who.int/news-room/fact-sheets/detail/sanitation.

[4] Fagunwa OE, Mthiyane T, Fagunwa A, Olayemi KI, Alozie A, Onyeaka H, et al. Priority regions for eliminating open defecation in Africa: implications for antimicrobial resistance. Environ Dev Sustain. 2023. doi: 10.1007/s10668-023-03992-6

[5] CDC—Centers for Disease Control and Prevention. Global Water, Sanitation and Hygiene Home—Healthy Water. 2017 [cited 23 Feb 2020]. Available: http://www.cdc.gov/healthywater/global/.

[6] WHO. Sanitation. 21 Mar 2022 [cited 24 Mar 2022]. Available: https://www.who.int/news-room/fact-sheets/detail/sanitation.

[7] ReliefWeb. Sanitation Fact sheet, Reviewed November 2016—World. 2016 [cited 24 Mar 2022]. Available: https://reliefweb.int/report/world/sanitation-fact-sheet-reviewed-november-2016.

[8] World Health Organization. Burden of disease attributable to unsafe drinking water, sanitation, and hygiene, 201 9 U p d ate. Geneva, Switzerland; 2023. Available: https://www.who.int/activities/estimating-WASH-related-burden-of-disease.

[9] WHO. Sanitation. In: WHO Press [Internet]. 2019 [cited 23 Feb 2020]. Available: https://www.who.int/news-room/fact-sheets/detail/sanitation.

[10] Freeman MC, Majorin F, Boisson S, Routray P, Torondel B, Clasen T. The impact of a rural sanitation programme on safe disposal of child faeces: A cluster randomised trial in Odisha, India. Trans R Soc Trop Med Hyg. 2016;110: 386–392. doi: 10.1093/trstmh/trw043 27496512 PMC5916378

[11] Sara S, Graham J. Ending open defecation in rural Tanzania: Which factors facilitate latrine adoption? Int J Environ Res Public Health. 2014;11: 9854–9870. doi: 10.3390/ijerph110909854 25247427 PMC4199054

[12] Majorin F, Freeman MC, Barnard S, Routray P, Boisson S, Clasen T. Child feces disposal practices in rural Orissa: A cross sectional study. PLoS One. 2014;9: 1–7. doi: 10.1371/journal.pone.0089551 24586864 PMC3930746

[13] Islam M, Ercumen A, Ashraf S, Rahman M, Shoab AK, Luby SP, et al. Unsafe disposal of feces of children <3 years among households with latrine access in rural Bangladesh: Association with household characteristics, fly presence and child diarrhea. PLoS One. 2018;13: 1–13. doi: 10.1371/journal.pone.0195218 29621289 PMC5886761

[14] Morita T, Godfrey S, George CM. Systematic review of evidence on the effectiveness of safe child faeces disposal interventions. Trop Med Int Heal. 2016;21: 1403–1419. doi: 10.1111/tmi.12773 27546207

[15] Azage M, Haile D. Factors associated with safe child feces disposal practices in Ethiopia: evidence from demographic and health survey. Arch Public Heal. 2015;73: 1–9. doi: 10.1186/s13690-015-0090-z 26504520 PMC4620604

[16] Phaswana-Mafuya N, Shukla N. Factors that could motivate people to adopt safe hygienic practices in the Eastern Cape Province, South Africa. Afr Health Sci. 2005;5: 21–28. 15843127 PMC1831901

[17] Alhaji A Aliyu TD. Factors associated with safe disposal practices of childs feaces in Nigeria: Evidence from 2013 Nigeria demographic and health survey. Niger Med J. 2019;60: 198–204. 31831940 10.4103/nmj.NMJ_3_19 PMC6892328

[18] Page MJ, McKenzie JE, Bossuyt PM, Boutron I, Hoffmann TC, Mulrow CD, et al. Updating guidance for reporting systematic reviews: development of the PRISMA 2020 statement. J Clin Epidemiol. 2021;134: 103–112. doi: 10.1016/j.jclinepi.2021.02.003 33577987

[19] Moola S, Munn Z, Sears K, Sfetcu R, Currie M, Lisy K, et al. Conducting systematic reviews of association (etiology): The Joanna Briggs Institute’s approach. Int J Evid Based Healthc. 2015;13: 163–169. doi: 10.1097/XEB.0000000000000064 26262566

[20] Aromataris E, Munn Z. JBI Reviewer’s Manual. JBI Rev Man. 2020. doi: 10.46658/jbirm-19-01

[21] UNICEF/WHO. Core questions on water, sanitation and hygiene for household surveys: 2018 update. New York; 2018. pp. 1–24. Available: https://washdata.org.

[22] Higgins JPT, Thompson SG, Deeks JJ, Altman DG. Measuring inconsistency in meta-analyses. BMJ. 2003;327: 557–560. doi: 10.1136/bmj.327.7414.557 12958120 PMC192859

[23] Beardsley R, Cronk R, Tracy W, Fleming L, Ng’ambi M, Tidwell JB, et al. Factors associated with safe child feces disposal in Ethiopia, India, and Zambia. Int J Hyg Environ Health. 2021;237: 113832. doi: 10.1016/j.ijheh.2021.113832 34454254

[24] Sahiledengle B. Prevalence and associated factors of safe and improved infant and young children stool disposal in Ethiopia: Evidence from demographic and health survey. BMC Public Health. 2019;19: 1–13. doi: 10.1186/s12889-019-7325-9 31331313 PMC6647302

[25] Aluko OO, Afolabi OT, Olaoye EA, Adebayo AD, Oyetola SO, Abegunde OO. The management of the faeces passed by under five children: an exploratory, cross-sectional research in an urban community in Southwest Nigeria. BMC Public Health. 2017;17: 1–15. doi: 10.1186/s12889-017-4078-1 28178955 PMC5299761

[26] Aliyu A, Dahiru T. Factors associated with safe disposal practices of child’s faeces in Nigeria: Evidence from 2013 Nigeria demographic and health survey. Niger Med J. 2019;60: 198. doi: 10.4103/nmj.NMJ_3_19 31831940 PMC6892328

[27] Sahiledengle B. Unsafe child feces disposal status in Ethiopia: What factors matter? Analysis of pooled data from four demographic and health surveys. BMC Public Health. 2020;20: 1–12. doi: 10.1186/s12889-020-08945-6 32460735 PMC7254708

[28] Nkoka O. Correlates of appropriate disposal of children’s stools in Malawi: A multilevel analysis. BMC Public Health. 2020;20: 1–10. doi: 10.1186/s12889-020-08725-2 32357929 PMC7195806

[29] Simelane MS, Chemhaka GB, Maphosa T, Zwane E. Unsafe disposal of faeces and its correlates among children under three years in Eswatini. South African J Child Heal. 2020;14: 217–223. doi: 10.7196/SAJCH.2020.v14i4.1726

[30] Seidu AA, Ahinkorah BO, Kissah-Korsah K, Agbaglo E, Dadzie LK, Ameyaw EK, et al. A multilevel analysis of individual and contextual factors associated with the practice of safe disposal of children’s faeces in sub-Saharan Africa. PLoS One. 2021;16: 1–17. doi: 10.1371/journal.pone.0254774 34339451 PMC8328335

[31] Tsegaw M, Mulat B, Shitu K. Safe stool disposal and associated factors among mothers of children under-two age in Gambia: Evidence from Gambia Demographic Health Survey. PLoS One. 2023;18: 1–11. doi: 10.1371/journal.pone.0284986 37126505 PMC10150983

[32] Demissie GD, Zerihun MF, Ekubagewargies DT, Yeshaw Y, Jemere T, Misganaw B, et al. Associated factors of safe child feces disposal in sub-Saharan Africa: Evidence from recent demographic and health surveys of 34 sub-Saharan countries. PLoS One. 2023;18: 1–11. doi: 10.1371/journal.pone.0281451 36758034 PMC9910663

[33] Addis M, Worku W, Bogale L, Shimelash A, Tegegne E. Hygienic Child Feces Disposal Practice and Its Associated Factors among Mothers/Caregivers of Under Five Children in West Armachiho District, Northwest Ethiopia. Environ Health Insights. 2022;16. doi: 10.1177/11786302221114738 35910283 PMC9335496

[34] Seidu AA. Are children’s stools in Ghana disposed of safely? Evidence from the 2014 Ghana demographic and health survey. BMC Public Health. 2021;21: 1–10. doi: 10.1186/s12889-021-10155-7 33422043 PMC7797132

[35] Ayele Y, Yemane D, Redae G, Mekibib E. Child feces disposal practice and associated factors: A dilemma in Tigray, northern Ethiopia. J Water Sanit Hyg Dev. 2018;8: 62–70. doi: 10.2166/washdev.2017.129

[36] Soboksa NE, Gar SR, Hailu AB, Alemu BM. Child defecation, feces disposal practices and associated factors in community-led total sanitation adopted districts in Jimma Zone, Ethiopia. Environ Challenges. 2021;3: 100059. doi: 10.1016/j.envc.2021.100059

[37] Kasye DG, Garoma NH, Kassa MA. Assessment of the Prevalence of Diarrheal Disease Under-five Children Serbo Town, Jimma Zone South West Ethiopia. Clin Mother Child Heal. 2018;15: 1–6. doi: 10.4172/2090-7214.1000281

[38] Prüss-Ustün A, Bartram J, Clasen T, Colford JM, Cumming O, Curtis V, et al. Burden of disease from inadequate water, sanitation and hygiene in low- and middle-income settings: A retrospective analysis of data from 145 countries. Trop Med Int Heal. 2014;19: 894–905. doi: 10.1111/tmi.12329 24779548 PMC4255749

[39] WHO. Sanitation Key facts. In: WHO [Internet]. 2023 [cited 22 Oct 2022]. Available: https://www.who.int/news-room/fact-sheets/detail/sanitation.

[40] Bartram J, Cairncross S. Hygiene, sanitation, and water: Forgotten foundations of health. PLoS Med. 2010;7. doi: 10.1371/journal.pmed.1000367 21085694 PMC2976722

[41] Ziegelbauer K, Speich B, Mäusezahl D, Bos R, Keiser J, Utzinger J. Effect of sanitation on soil-transmitted helminth infection: Systematic review and meta-analysis. PLoS Medicine. 2012. doi: 10.1371/journal.pmed.1001162 22291577 PMC3265535

[42] UNICEF & WHO. Progress on household drinking water, sanitation and hygiene 2000–2017: Special focus on inequalities. UNICEF WHO. 2019. Available: https://data.unicef.org/resources/progress-drinking-water-sanitation-hygiene-2019/%aA0https://www.eea.europa.eu/publications/industrial-waste-water-treatment-pressures%0Ahttp://files/558/Rapport EEA Industrial waste water treatment–pressures on Europe’s.

[43] Sprouse L, Liles A, Cronk R, Bauza V, Tidwell JB, Manga M. Interventions to address unsafe child feces disposal practices in the Asia-Pacific region: a systematic review. H2Open J. 2022;5: 583–602. doi: 10.2166/h2oj.2022.137

[44] Wolf J, Hubbard S, Brauer M, Ambelu A, Arnold BF, Bain R, et al. Effectiveness of interventions to improve drinking water, sanitation, and handwashing with soap on risk of diarrhoeal disease in children in low-income and middle-income settings: a systematic review and meta-analysis. Lancet. 2022;400: 48–59. doi: 10.1016/S0140-6736(22)00937-0 35780792 PMC9251635

[45] Gil A, Lanata C, Kleinau E, Penny M. Children’s Feces Disposal Practices in Developing Countries and Interventions to Prevent Diarrheal Diseases: A Literature Review. Environ Heal Proj. 2004. Available: http://pdf.usaid.gov/pdf_docs/PNACY780.pdf.

[46] Bauza V, Majorin F, Routray P, Sclar GD, Caruso BA, Clasen T. Child feces management practices and fecal contamination: A cross-sectional study in rural Odisha, India. Sci Total Environ. 2020;709: 136169. doi: 10.1016/j.scitotenv.2019.136169 31905545 PMC7031693

