# Supplementary material for: Does owning improved latrine facilities enhance the safe disposal of child feces in Africa? a systematic review and meta-analysis
Source: PLoS One. 2024 May 16;19(5):e0303754. doi: 10.1371/journal.pone.0303754 (PMC11098413; doi:10.1371/journal.pone.0303754)
Supplement: S1 Table — (DOCX) [file pone.0303754.s002.docx]

Table S1. Search strategy for the PubMed database

| S /No | Search set PubMed databases |  |
| --- | --- | --- |
|  | (("sanitation"[MeSH Terms] OR "toilet facilities"[MeSH Terms] OR Improved latrine facilities [tw])) | 119,322 |
|  | "ownership"[MeSH Terms] OR "ownership"[tw] OR "ownerships"[tw] OR "presence"[tw] OR "presences"[tw] OR "halve"[All Fields] OR "halved"[tw] OR "halves"[tw] OR "halving"[tw] | 1,991,639 |
|  | "Feces"[MeSH] OR "feces"[tw] OR "faeces"[tw] OR"faecal"[tw] OR "fecal"[tw] OR "excreta"[tw] OR "stool"[tw] OR "stools"[tw] | 235,234 |
|  | dispos*[tw] OR "excrement"[tw] OR "defecation"[tw] OR manag*[tw] OR remov*[tw] OR "Waste Management"[MeSH] | 2,878,714 |
|  | "babies"[tw] OR "baby"[tw] OR "boy"[tw] OR "boys"[tw] OR "Child, Preschool"[MeSH] OR "Child"[MeSH] OR child*[tw] OR "girl"[tw] OR "girls"[tw] OR infan*[tw] OR "Infant, Newborn"[MeSH] OR "Infant"[MeSH] OR "kid"[tw] OR "kids"[tw] OR "less than 5"[tw] OR "less than five"[tw] OR neonat*[tw] OR new-born*[tw] OR newborn*[tw] OR "pediatric"[tw] OR pre school*[tw] OR preschool*[tw] OR toddler*[tw] OR "under 5"[tw] OR "under five"[tw] OR "under fives"[tw] | 3,824,695 |
|  | 1 AND 2 AND 3 AND 4 AND 5 | 71 |
|  | "africa"[MeSH Terms] OR "africa"[tw] OR "africa s"[tw] OR "africas"[tw] | 480,450 |
|  | 6 AND 7 | 40 |

MeSH = Medical subject headings, tw = text word
