# Supplementary material for: Does owning improved latrine facilities enhance the safe disposal of child feces in Africa? a systematic review and meta-analysis
Source: PLoS One. 2024 May 16;19(5):e0303754. doi: 10.1371/journal.pone.0303754 (PMC11098413; doi:10.1371/journal.pone.0303754)
Supplement: S2 Table — (DOCX) [file pone.0303754.s003.docx]

**Table S2:**  **Does owning improved latrine facilities enhance the safe disposal of child feces in Africa? A systematic review and meta-analysis**

Correspondence: [yeroosaa@gmail.com](mailto:yeroosaa@gmail.com)

| No. | Author, publication year | Eligibility criteria | Study subjects and the setting | Exposure measured in a valid and reliable way 'gold standard' | A specified diagnosis or definition | Confounding factors | Dealing with confounding factors | Outcomes measured in a valid and reliable way | Appropriate statistical analysis | Scores (8) | Quality (high, moderate, low) |
| --- | --- | --- | --- | --- | --- | --- | --- | --- | --- | --- | --- |
| 1 | (Ayele et al. 2018) | Yes | Yes | Yes | No | No | NA | Yes | Yes | 6 | Moderate |
| 2 | (Addis et al. 2022) | Yes | Yes | Yes | Yes | No | NA | Yes | Yes | 7 | High |
| 3 | (Soboksa et al. 2021) | Yes | Yes | Yes | Yes | No | NA | Yes | Yes | 7 | High |
| 4 | (Aluko et al. 2017) | Yes | Yes | Yes | Yes | No | NA | No | Yes | 6 | Moderate |
| 5 | (Beardsley et al. 2021) | Yes | Yes | Yes | Yes | No | NA | Yes | Yes | 7 | High |
| 6 | (Sahiledengle 2019) | Yes | Yes | Yes | Yes | No | NA | Yes | Yes | 7 | High |
| 7 | (Azage and Haile 2015) | Yes | Yes | Yes | Yes | No | NA | Yes | Yes | 7 | High |
| 8 | (Aliyu and Dahiru 2019) | Yes | Yes | Yes | Yes | No | NA | Yes | Yes | 7 | High |
| 9 | (Sahiledengle 2020) | Yes | Yes | Yes | Yes | No | NA | Yes | Yes | 7 | High |
| 10 | (Nkoka 2020) | Yes | Yes | Yes | Yes | No | NA | Yes | Yes | 7 | High |
| 11 | (Simelane et al. 2020) | Yes | Yes | Yes | Yes | No | NA | Yes | Yes | 7 | High |
| 12 | (Seidu et al. 2021) | Yes | Yes | Yes | Yes | No | NA | Yes | Yes | 7 | High |
| 13 | (Tsegaw et al. 2023) | Yes | Yes | Yes | Yes | No | NA | Yes | Yes | 7 | High |
| 14 | (Demissie et al. 2023) | Yes | Yes | Yes | Yes | No | NA | Yes | Yes | 7 | High |
| 15 | (Seidu 2021) | Yes | Yes | Yes | Yes | No | NA | Yes | Yes | 7 | High |

**REFERENCES**

Addis M, Worku W, Bogale L, Shimelash A, Tegegne E. Hygienic Child Feces Disposal Practice and Its Associated Factors among Mothers/Caregivers of Under Five Children in West Armachiho District, Northwest Ethiopia. Environ Health Insights. 2022;16.

Aliyu A, Dahiru T. Factors associated with safe disposal practices of child’s faeces in Nigeria: Evidence from 2013 Nigeria demographic and health survey. Nigerian Medical Journal. 2019;60(4):198.

Aluko OO, Afolabi OT, Olaoye EA, Adebayo AD, Oyetola SO, Abegunde OO. The management of the faeces passed by under five children: an exploratory, cross-sectional research in an urban community in Southwest Nigeria. BMC Public Health. 2017;17(1):1–15.

Ayele Y, Yemane D, Redae G, Mekibib E. Child feces disposal practice and associated factors: A dilemma in Tigray, northern Ethiopia. Journal of Water Sanitation and Hygiene for Development. 2018;8(1):62–70.

Azage M, Haile D. Factors associated with safe child feces disposal practices in Ethiopia: evidence from demographic and health survey. Archives of Public Health. 2015;73(40):1–9.

Beardsley R, Cronk R, Tracy W, Fleming L, Ng’ambi M, Tidwell JB, et al. Factors associated with safe child feces disposal in Ethiopia, India, and Zambia. Int J Hyg Environ Health. 2021;237(August):113832.

Demissie GD, Zerihun MF, Ekubagewargies DT, Yeshaw Y, Jemere T, Misganaw B, et al. Associated factors of safe child feces disposal in sub-Saharan Africa: Evidence from recent demographic and health surveys of 34 sub-Saharan countries. PLoS One. 2023;18(2 February):1–11.

Nkoka O. Correlates of appropriate disposal of children’s stools in Malawi: A multilevel analysis. BMC Public Health. 2020;20(1):1–10.

Phaswana-Mafuya N, Shukla N. Factors that could motivate people to adopt safe hygienic practices in the Eastern Cape Province, South Africa. Afr Health Sci. 2005;5(1):21–8.

Sahiledengle B. Prevalence and associated factors of safe and improved infant and young children stool disposal in Ethiopia: Evidence from demographic and health survey. BMC Public Health. 2019;19(1):1–13.

Sahiledengle B. Unsafe child feces disposal status in Ethiopia: What factors matter? Analysis of pooled data from four demographic and health surveys. BMC Public Health. 2020;20(1):1–12.

Seidu AA, Ahinkorah BO, Kissah-Korsah K, Agbaglo E, Dadzie LK, Ameyaw EK, et al. A multilevel analysis of individual and contextual factors associated with the practice of safe disposal of children’s faeces in sub-Saharan Africa. PLoS One. 2021;16(8 August):1–17.

Seidu AA. Are children’s stools in Ghana disposed of safely? Evidence from the 2014 Ghana demographic and health survey. BMC Public Health. 2021;21(1):1–10.

Simelane MS, Chemhaka GB, Maphosa T, Zwane E. Unsafe disposal of faeces and its correlates among children under three years in Eswatini. South African Journal of Child Health. 2020;14(4):217–23.

Soboksa NE, Gar SR, Hailu AB, Alemu BM. Child defecation , feces disposal practices and associated factors in community-led total sanitation adopted districts in Jimma Zone , Ethiopia. Environmental Challenges. 2021;3(February):100059.

Tsegaw M, Mulat B, Shitu K. Safe stool disposal and associated factors among mothers of children under-two age in Gambia : Evidence from Gambia Demographic Health Survey. PLoS One. 2023;18(5):1–11.
